# Enhanced High-Temperature (600 °C) NO_2_ Response of ZnFe_2_O_4_ Nanoparticle-Based Exhaust Gas Sensors

**DOI:** 10.3390/nano10112133

**Published:** 2020-10-27

**Authors:** Adeel Afzal, Adnan Mujahid, Naseer Iqbal, Rahat Javaid, Umair Yaqub Qazi

**Affiliations:** 1 Department of Chemistry, College of Science, University of Hafr Al Batin, P.O. Box 1803, Hafr Al Batin 39524, Saudi Arabia; naseeriqbal@uhb.edu.sa (N.I.); umair_qazi@yahoo.com (U.Y.Q.); 2 Institute of Chemistry, University of Punjab, Quaid-i-Azam Campus, Lahore 54590, Pakistan; adnan.mujahid@pu.edu.pk; 3 Renewable Energy Research Center, Fukushima Renewable Energy Institute, National Institute of Advanced Industrial Science and Technology, AIST, 2-2-9 Machiikedai, Koriyama, Fukushima 963-0298, Japan; rahat.javaid@aist.go.jp; 4 Division of Nanomaterials and Chemistry, Hefei National Laboratory for Physical Sciences at Microscale, University of Science and Technology of China, Hefei 230026, China

**Keywords:** annealing temperature, chemiresistors, gas sensors, oxygen vacancies, sensing mechanism, ZnFe_2_O_4_ nanoparticles

## Abstract

Fabrication of gas sensors to monitor toxic exhaust gases at high working temperatures is a challenging task due to the low sensitivity and narrow long-term stability of the devices under harsh conditions. Herein, the fabrication of a chemiresistor-type gas sensor is reported for the detection of NO_2_ gas at 600 °C. The sensing element consists of ZnFe_2_O_4_ nanoparticles prepared via a high-energy ball milling and annealed at different temperatures (600–1000 °C). The effects of annealing temperature on the crystal structure, morphology, and gas sensing properties of ZnFe_2_O_4_ nanoparticles are studied. A mixed spinel structure of ZnFe_2_O_4_ nanoparticles with a lattice parameter of 8.445 Å is revealed by X-ray diffraction analysis. The crystallite size and X-ray density of ZnFe_2_O_4_ nanoparticles increase with the annealing temperature, whereas the lattice parameter and volume are considerably reduced indicating lattice distortion and defects such as oxygen vacancies. ZnFe_2_O_4_ nanoparticles annealed at 1000 °C exhibit the highest sensitivity (0.13% ppm^–1^), sharp response (*τ_res_* = 195 s), recovery (*τ_rec_* = 17 s), and linear response to 100–400 ppm NO_2_ gas. The annealing temperature and oxygen vacancies play a major role in determining the sensitivity of devices. The plausible sensing mechanism is discussed. ZnFe_2_O_4_ nanoparticles show great potential for high-temperature exhaust gas sensing applications.

## 1. Introduction

Hazardous exhaust gases such as nitrogen dioxide (NO_2_) and sulfur dioxide (SO_2_) are the major atmospheric pollutants [1]. The European Union’s (E.U.) ambient air quality directives have set the hourly NO_2_ concentration threshold as 200 µg/m^3^ [2]. According to the European Environment Agency (EEA) report published in 2016, NO_2_ pollution was responsible for 71,000 premature deaths in the E.U. [3]. Thus, it is important to detect the emission and subsistence of NO_2_ in indoor and outdoor air. The main source of NO_2_ pollution is the exhaust emissions as a result of the combustion processes in motor vehicles and manufacturing industries [4]. The direct inspection of the exhaust emissions requires devices that can detect NO_2_ at high temperatures, i.e., usually ≥500 °C [5]. In this regard, metal oxide-based electronic gas sensors are the most sought-after devices for applications in harsh environments [6,7,8].

According to a recent review of high-temperature gas sensors, Ghosh et al. [9] noted the majority of the metal oxide-based gas sensors work at moderately high temperatures only, while the sensitivity of metal oxides is substantially influenced at temperatures above 350 °C. Albeit a large number of metal oxide-based NO_2_ gas sensors are reported [10,11,12,13], only a few work at high temperatures, i.e., ≥600 °C. For instance, Miura et al. [14] reported Yt-stabilized zirconia and spinel ZnFe_2_O_4_ sensing electrodes for the electrochemical detection of NO*_x_* at 600–700 °C. However, chemiresistive-type NO_2_ gas sensors for high-temperature applications are rarely reported [13,15,16]. Therefore, the fabrication of high-temperature NO_2_ gas sensors for harsh environments is highly desired due to their widespread applications in all types of combustion systems.

This article reports the first high-temperature thick film chemiresistive gas sensor for NO_2_ detection at 600 °C. The sensor is based on highly stable spinel zinc ferrite (ZnFe_2_O_4_) nanoparticles prepared via a solid-state, high-energy ball-milling (HEBM) process followed by high-temperature thermal annealing at different temperatures (600, 800, and 1000 °C). ZnFe_2_O_4_ nanoparticles have been used for the detection of toxic organic vapors and gases such as acetone at 260 [17] and 275 °C [18], ethanol at 27 [19] and 220 °C [20], toluene at 300 °C [21], H_2_S at 85 °C [22] and 135 °C [23], and O_2_ at 180 °C [24]. Recently, Runa et al. [25] fabricated a chemiresistive NO_2_ gas sensor using ZnO/ZnFe_2_O_4_ composites with p-n heterostructure, which revealed excellent selectivity and high gas response toward 0.1–20 ppm NO_2_ compared to pure ZnO. However, the gas response diminished rapidly at temperatures of ≥220 °C [25]. In this work, the effects of high-temperature annealing on the crystal structure and NO_2_ gas sensing properties are studied. The cubic spinel ZnFe_2_O_4_ nanoparticles are stable at high temperatures and demonstrate excellent NO_2_ sensing capability at 600 °C with fast response and recovery times.

## 2. Materials and Methods

Iron(III) oxide (Fe_2_O_3_ nanopowder) and zinc oxide (ZnO nanopowder) obtained from MilliporeSigma (Merck KGaA, Darmstadt, Germany) were used to prepare ZnFe_2_O_4_ nanoparticles. ZnFe_2_O_4_ nanoparticles were synthesized by high-energy ball milling (HEBM) process using a SPEX™ 8000M Mixer/Mill™ (SPEX^®^ SamplePrep, New Jersey, NJ, USA). The ball mill was equipped with a 500-cc stainless steel vessel containing stainless steel balls for mechanical milling of Fe_2_O_3_ and ZnO. The mass ratio of steel balls and chemical powders was fixed at 50:1. HEBM was performed under ambient conditions for 2 h at 600 rpm. The product was subsequently vacuum annealed at 600, 800, and 1000 °C for 2 h, and characterized. Corresponding to the annealing temperature (600–1000 °C), the samples were abbreviated as ZnFe_2_O_4_-600, ZnFe_2_O_4_-800, and ZnFe_2_O_4_-1000, respectively.

The crystal structure of the annealed ZnFe_2_O_4_ nanoparticles was studied with a STOE STADI P X-ray diffractometer (XRD) (STOE & Cie GmbH, Darmstadt, Germany) using a Cu Kα irradiation source (λ = 1.5406 Å). The samples were scanned in the 2θ range of 10°–90° with a scan rate of 2°/min. The crystallite size (*D*) is determined by the Scherrer’s formula [26] (*D = Kλ/Bcosθ*), where *K* is a numerical factor referred to as the crystallite-shape factor with an approximate value of 0.89, *λ* is the wavelength of the X-rays, *B* is full-width at half-maximum of the most intense (311) diffraction peak in radians, and *θ* is the Bragg angle. The experimental lattice parameter (*a*), X-ray density (*ρ_xrd_*), and the specific surface area (*S_A_*) are also calculated from the XRD data of annealed ZnFe_2_O_4_ nanoparticles, as described elsewhere [27,28].

The microstructure and surface morphology of ZnFe_2_O_4_ nanoparticles were studied with a JEOL JSM-6510 scanning electron microscope (SEM) (JEOL Ltd., Tokyo, Japan). The elemental composition of ZnFe_2_O_4_ nanoparticles was determined with the energy-dispersive X-ray spectroscopy (EDS) (JEOL Ltd., Tokyo, Japan).

Thick-film chemiresistor-type gas sensors were fabricated by mixing an appropriate amount of ZnFe_2_O_4_-600, ZnFe_2_O_4_-800, and ZnFe_2_O_4_-1000 nanoparticles with absolute ethanol to make a thick slurry, which was subsequently drop-coated onto alumina micro-hotplates with vapor-deposited platinum (Pt) contacts. The devices were placed in a vacuum oven at 80 °C for 2 h to dry and stabilize the sensing element. The chemiresistor-type devices were installed in a gas sensing chamber fitted with the electrical connections and the gas inlet and outlet. The measurements were performed with a Keithley 6517A electrometer. The sensor responses were measured simultaneously at 600 °C for 100–400 ppm of NO_2_ gas. The sensor response (*S*) is defined as *S(%) = (R_g_ – R_a_) ×* 100*/R_a_*, where *R_a_* and *R_g_* are the resistances in air and (100–400 ppm) NO_2_ gas.

## 3. Results and Discussion

**Figure 1:**
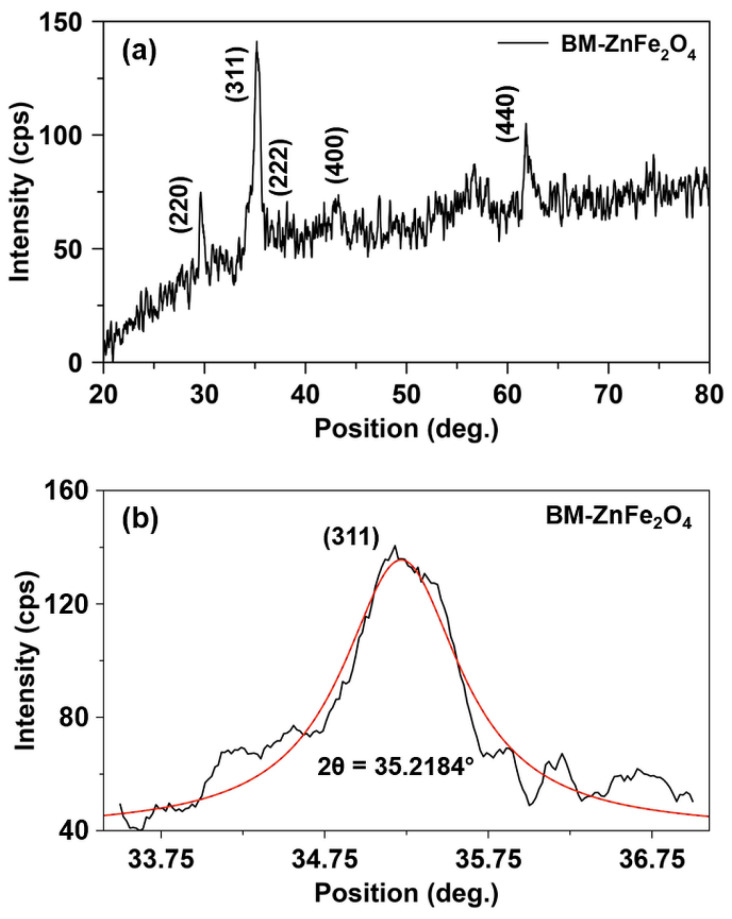
(**a**) X-ray diffraction pattern of the as-synthesized, ball-milled zinc ferrite (BM-ZnFe_2_O_4_) nanoparticles. (**b**) The characteristic (311) plane diffraction of cubic spinel BM-ZnFe_2_O_4_ nanoparticles.

Figure 1 shows the XRD pattern of as-synthesized ball-milled ZnFe_2_O_4_ nanoparticles, referred to as BM-ZnFe_2_O_4_. The HEBM process yields crystalline BM-ZnFe_2_O_4_ nanoparticles with a cubic spinel lattice structure as indicated by the presence of characteristic (311) diffraction at 35.22° (2θ) position. The crystallite size (*D*) of as-synthesized BM-ZnFe_2_O_4_ nanoparticles is 9.30 nm. The lattice parameter (*a*) is calculated to be 8.445 Å, which is in agreement with the values reported for spinel ZnFe_2_O_4_ nanostructures in the literature and the standard value of bulk ZnFe_2_O_4_ (*a* = 8.441 Å) [29,30,31]. The lattice parameter of as-synthesized BM-ZnFe_2_O_4_ nanoparticles is slightly higher (~0.05%) than the standard value that may be inherent to the ball-milling process because an increase in the lattice parameter of ball-milled ZnFe_2_O_4_ samples has been reported earlier [32,33,34].

Theoretically, the cation distribution in a perfect normal spinel ZnFe_2_O_4_ unit cell is (Zn^2+^)_tet_[Fe^3+^]_oct_O_4_, i.e., the tetrahedral (A) and octahedral (B) sites are solely occupied by Zn^2+^ and Fe^3+^ cations, respectively [35]. However, in nanocrystalline ZnFe_2_O_4_ the contrary distributions of Zn^2+^ and Fe^3+^ cations on both A and B sites are observed [36,37], which form mixed (or random) spinel structure. According to Chinnasamy et al. [32], the slight increase in the lattice parameter is attributed to the lattice expansion caused by the occupation of B sites by Zn^2+^ ions. Thus, the XRD pattern of as-synthesized BM-ZnFe_2_O_4_ nanoparticles indicates the formation of a mixed cubic spinel lattice. Nonetheless, BM-ZnFe_2_O_4_ nanoparticles are annealed at different temperatures to examine the effect of annealing on the crystal structure evolution, lattice parameter, crystallite size, and morphology of ZnFe_2_O_4_ nanoparticles.

**Figure 2:**
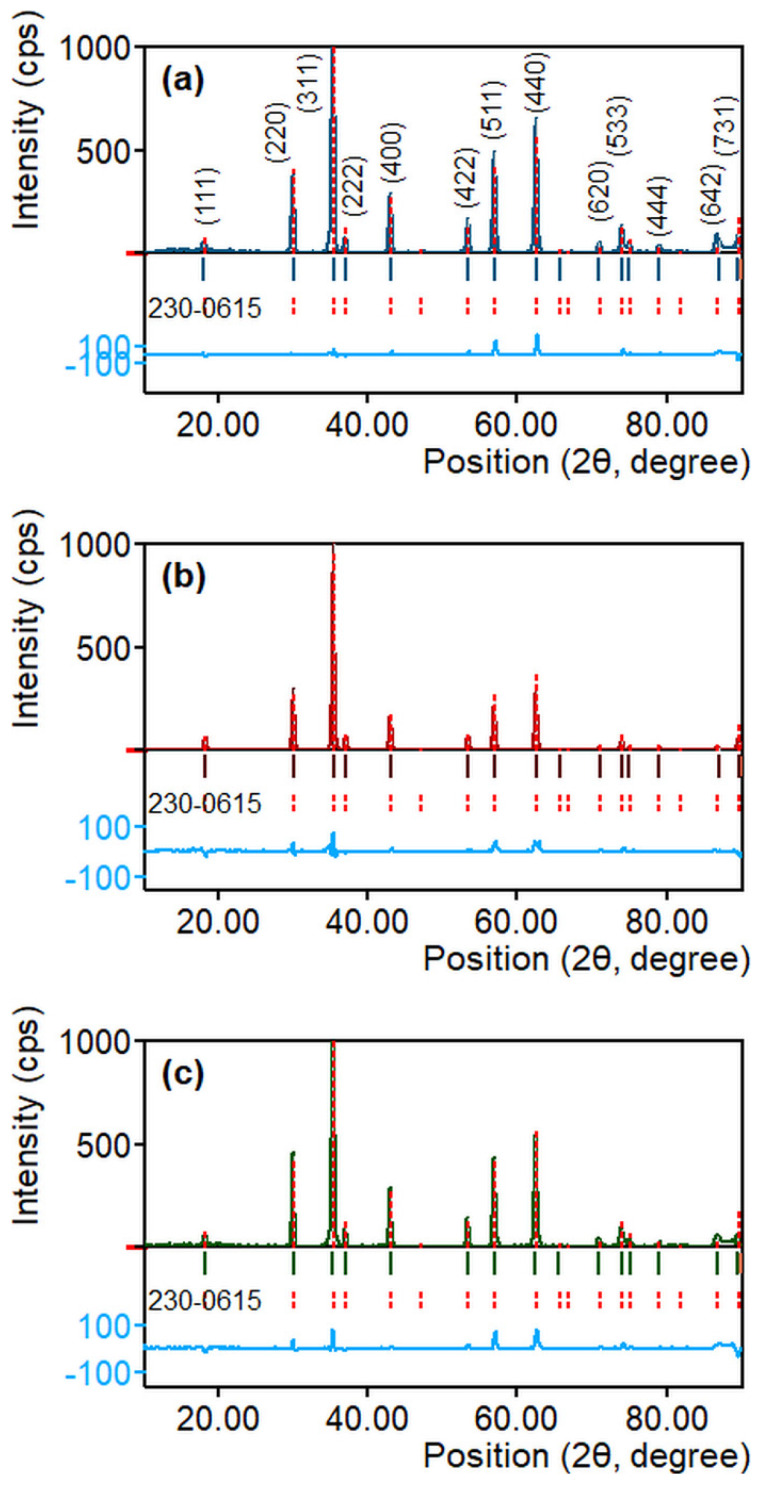
X-ray diffraction patterns of ZnFe_2_O_4_ nanoparticles annealed at different temperatures: (**a**) 600, (**b**) 800, and (**c**) 1000 °C.

High-temperature annealing is an important step in the fabrication of ZnFe_2_O_4_ nanoparticles, as it renders stability and improves the physical properties of ZnFe_2_O_4_ [38,39]. Figure 2 shows the XRD patterns of ZnFe_2_O_4_ nanoparticles annealed at 600, 800, and 1000 °C for 2 h. XRD patterns are refined using Match! (version 3.11.1.183) and FullProf programs for phase identification from X-ray powder diffraction. All samples exhibit a crystalline structure with the characteristic diffractions corresponding to the following miller indices: (111), (220), (311), (222), (400), (422), (511), (440), (620), and (533), which conform to the crystallography open database card number 230–0615 [40]. XRD results substantiate the formation of the cubic spinel ferrite structure with the *Fd-3m* space group. Also, the XRD patterns align well with the reported literature for ZnFe_2_O_4_ nanoparticles [30,41,42]. The absence of additional diffraction peaks corresponding to the impurities or unreacted oxides also reveals the formation of a single-phase cubic spinel lattice [40].

**Figure 3:**
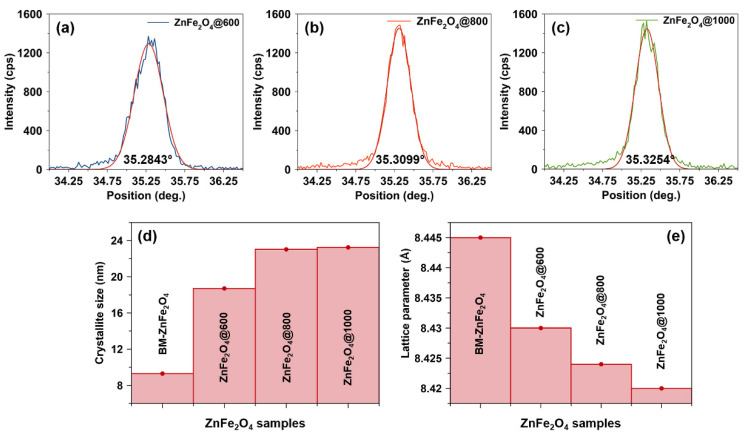
The characteristic (311) plane diffractions of cubic spinel ZnFe_2_O_4_ nanoparticles annealed at (**a**) 600, (**b**) 800, and (**c**) 1000 °C. (**d**) The crystallite size, and (**e**) lattice parameter of ZnFe_2_O_4_ nanoparticles as a function of annealing temperature.

Figure 3a–c shows the most intense diffractions of the characteristic (311) plane in ZnFe_2_O_4_ nanoparticles annealed at different temperatures. The XRD data were used to calculate the crystallite size (*D*), lattice parameter (*a*), interplanar distance (*d_311_*), volume (*V*), X-ray density (*ρ_xrd_*), and specific surface area (*S*) of the annealed ZnFe_2_O_4_ nanoparticles. Table 1 presents these structural parameters for different samples. The effect of annealing is obvious because of the changes in position and breadth of (311) diffraction peak as a function of the annealing temperature, which reveal variations in the crystallite size and lattice parameter. The position of (311) shifts to a higher 2θ value as the annealing temperature increases.

As shown in Figure 3d, the crystallite size of ZnFe_2_O_4_ nanoparticles increases with the annealing time, which is certainly comprehensible because annealing results in grain growth, and the higher the temperature, the greater is the crystallite size [43,44,45]. The annealing at 600 °C doubles the crystallite size of ZnFe_2_O_4_@600 nanoparticles compared to as-synthesized BM-ZnFe_2_O_4_ nanoparticles. While annealing at 800 °C results in a further increase in the crystallite size, the crystallite sizes of ZnFe_2_O_4_@800 and ZnFe_2_O_4_@1000 nanoparticles are comparable. Thus, the little difference in the crystallites sizes of samples treated at 800 and 1000 °C means the rate or degree of annealing decreases with the increasing crystallite size [46].

On the other hand, the lattice parameter decreases with the increase in annealing temperature, as shown in Figure 3e. Although this is contrary to the findings reported earlier that demonstrate an increase in the lattice parameter upon high-temperature annealing [47,48,49], the lattice parameter and crystal structure essentially depend on the processing method and conditions. As discussed above, in the starting sample, as-synthesized BM-ZnFe_2_O_4_ nanoparticles exhibit a random spinel structure with a certain degree of inversion that is inherently observed for the ball-milled ZnFe_2_O_4_ nanoparticles [32,33]. The reduction in the lattice parameter of annealed ZnFe_2_O_4_ nanoparticles can be explained by the redistribution of cations and crystal defects (oxygen vacancies).

During the high-temperature annealing process, both Zn^2+^ and Fe^3+^ cations may alter positions that influence the crystal structure. For instance, Lemine et al. [50] demonstrated that a decrease in the lattice parameter (from 8.448 to 8.427 Å) was caused by the redistribution of cations within the interstitial sites. However, this can also be attributed to the crystal defects [39,51]. It is a well-known fact that high-temperature annealing induces lattice defects and distortions. Furthermore, in the case of nanocrystalline ZnFe_2_O_4_, it is believed that Zn^2+^ ions due to their volatile nature escape from the lattice during thermal treatment that successively results in oxygen vacancies [39,52]. Thus, a decrease in the lattice parameter (from 8.445 Å for BM-ZnFe_2_O_4_ to 8.420 Å for ZnFe_2_O_4_@1000) is attributed to the cationic redistribution (distortion) and lattice compression caused by escaping Zn^2+^ ions and oxygen vacancies.

Consequently, the interplanar distance and the volume of the annealed ZnFe_2_O_4_ nanoparticles are reduced as a function of the annealing temperature. On the other hand, the X-ray density increases (from 5.321 g cm^–3^ for BM-ZnFe_2_O_4_ to 5.368 g cm^–3^ for ZnFe_2_O_4_@1000) with the increase in annealing temperature. However, as shown in Table 1, the specific surface area is reduced to 48 m^2^ g^–1^ due to an increase in the crystallite size of the annealed ZnFe_2_O_4_ nanoparticles. These results demonstrate that ZnFe_2_O_4_@1000 and ZnFe_2_O_4_@800 nanoparticles have bigger crystallite size and smaller specific surface area, but the greatest number of defect sites (as oxygen vacancies) and a geometrically frustrated [53] or distorted cubic spinel crystal structure compared to as-synthesized BM-ZnFe_2_O_4_ nanoparticles.

**Figure 4:**
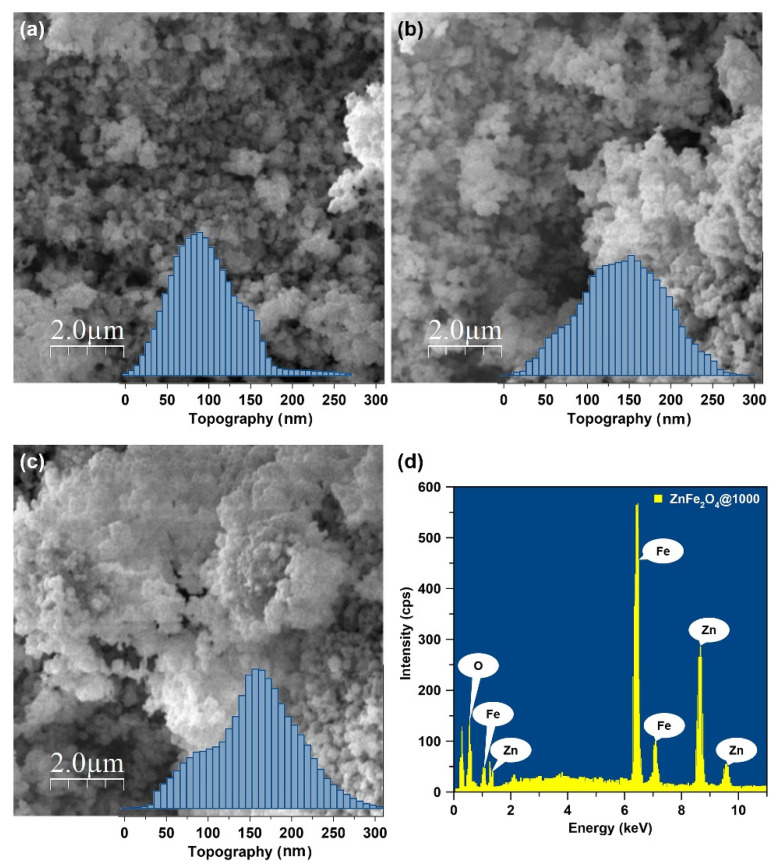
Scanning electron microscopy images of ZnFe_2_O_4_ nanoparticles annealed at (**a**) 600, (**b**) 800, and (**c**) 1000 °C. The respective histograms are given in the inset. (**d**) Energy-dispersive X-ray spectrum of ZnFe_2_O_4_ nanoparticles annealed at 1000 °C.

Figure 4a–c shows the SEM images of ZnFe_2_O_4_ nanoparticles annealed at different temperatures. An increase in the annealing temperature (to 1000 °C) results in a more compact surface, as shown in Figure 4c: the micrograph of ZnFe_2_O_4_@1000 nanoparticles. On the other hand, ZnFe_2_O_4_@600 nanoparticles annealed at 600 °C (Figure 4a) show less compact surface morphology with smaller particle size and relatively less aggregation of nanoparticles into clusters. The ZnFe_2_O_4_@800 nanoparticles demonstrate a similar surface morphology with slightly larger aggregates of nanoparticles, as shown in Figure 4b.

The image analysis of the scanning electron micrographs (via WSxM freeware [54]) shows the size distribution of ZnFe_2_O_4_ nanoparticles and the respective histograms are presented as insets in Figure 4a–c. ZnFe_2_O_4_@600 nanoparticles exhibit narrow size distribution with an average aggregate size of 100.2 nm, while ZnFe_2_O_4_@800 and ZnFe_2_O_4_@1000 nanoparticles reveal a relatively broad size distribution and an average aggregate size of 143.8 and 146.5 nm, respectively.

**Figure 5:**
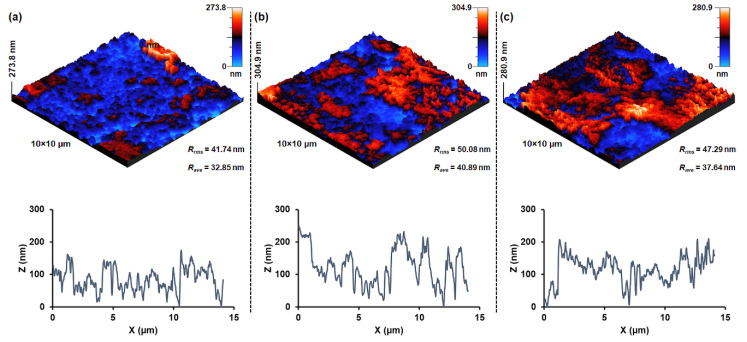
Three-dimensional micrographs and surface profiles showing the surface morphology, roughness, and topography of the sensitive element–ZnFe_2_O_4_ nanoparticles annealed at (**a**) 600, (**b**) 800, and (**c**) 1000 °C.

Figure 5 shows three-dimensional surface micrographs and topographic profiles of the sensing layers composed of ZnFe_2_O_4_ nanoparticles annealed at different temperatures. ZnFe_2_O_4_@600 surface exhibits a relatively smooth profile and roughness (Figure 5a). On the other hand, ZnFe_2_O_4_@800 (Figure 5b) and ZnFe_2_O_4_@1000 (Figure 5c) nanoparticles demonstrate higher roughness, greater particle size, and cluster formation. Thus, both X-ray diffraction and microscopic results indicate that ZnFe_2_O_4_ nanoparticles annealed at 800 and 1000 °C exhibit bigger crystallite size and a compact surface microstructure compared to those annealed at 600 °C.

Table 2 presents the elemental composition of the annealed ZnFe_2_O_4_ nanoparticles. Compared to theoretically calculated values (wt.% or at.%), annealed ZnFe_2_O_4_ nanoparticles exhibit variations. As the annealing temperature increases, the relative percentage of Fe increases while the proportions of Zn and O decrease. A decrease in the oxygen content with increasing temperature is attributed to the oxygen vacancies and lattice defects. In addition, the atomic ratio of Fe/Zn is found to be 2.07, 2.20, and 2.21 for ZnFe_2_O_4_@600, ZnFe_2_O_4_@800, and ZnFe_2_O_4_@1000 nanoparticles, respectively. The increase in the Fe/Zn ratio as a function of annealing temperature may be attributed to the volatile nature of Zn^2+^ ions [39], as discussed earlier. Thus, the results are consistent and exhibit the microstructure evolution in the annealed ZnFe_2_O_4_ nanoparticles as a function of annealing temperature.

**Figure 6:**
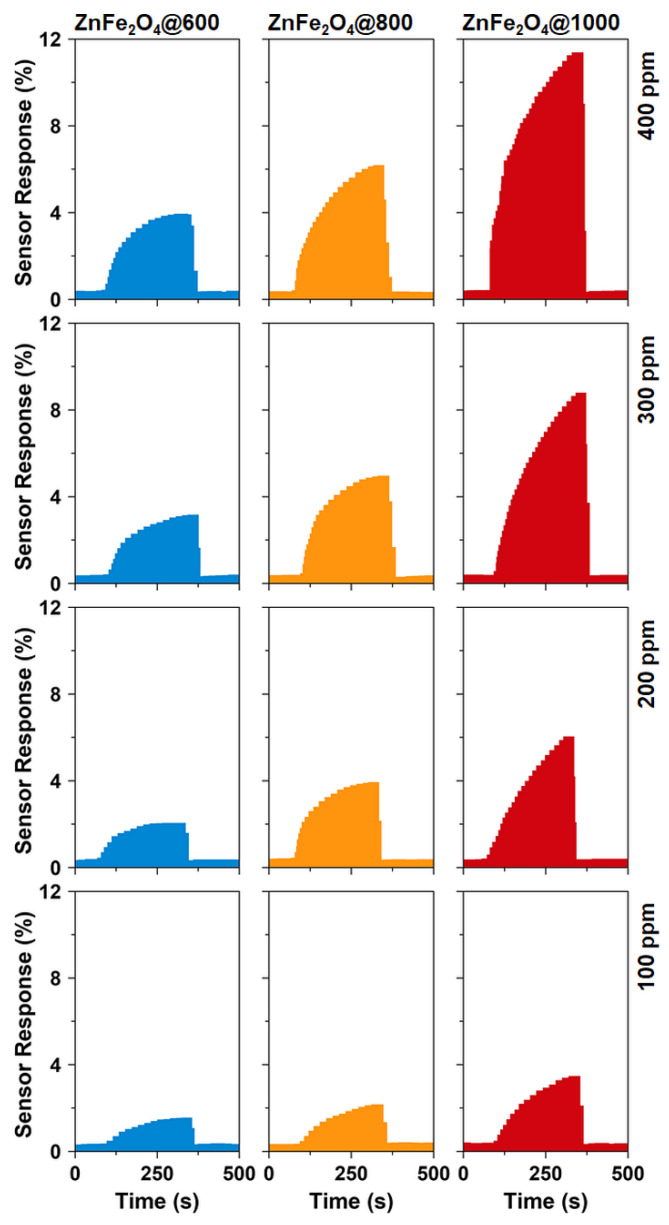
Time-dependent sensor response of the annealed ZnFe_2_O_4_ nanoparticles toward 100–400 ppm NO_2_ gas at 600 °C.

Figure 6 demonstrates the NO_2_ gas response of ZnFe_2_O_4_ nanoparticles annealed at different temperatures. The sensor measurements are performed at 600 °C. The as-synthesized BM-ZnFe_2_O_4_ nanoparticles based chemiresistive devices are not stable at 600 °C and do not show a measurable response to NO_2_ gas. On the other hand, all the annealed ZnFe_2_O_4_ samples show a significant measurable response to 100–400 ppm NO_2_, as shown in Figure 6. The sensor responses are generally saturated after ~4 min of exposure to the different concentrations of NO_2_ gas. ZnFe_2_O_4_@1000 nanoparticles exhibit the highest NO_2_ gas response, which is attributed to their greater stability at elevated temperatures and the presence of a large number of lattice defects. ZnFe_2_O_4_@600 and ZnFe_2_O_4_@800 nanoparticles also exhibit significant gas response at 600 °C. Peng et al. [55] recently demonstrated that the gas sensing properties of ZnFe_2_O_4_ nanoparticles could be enhanced by controlling the oxygen vacancies and that ZnFe_2_O_4_ nanoparticles with more oxygen vacancies revealed superior gas (acetone vapors) sensing performance at 280 °C. Thus, the higher NO_2_ response of ZnFe_2_O_4_@1000 nanoparticles may be attributed to the oxygen vacancies resulting from high-temperature annealing of nanoparticles.

**Figure 7:**
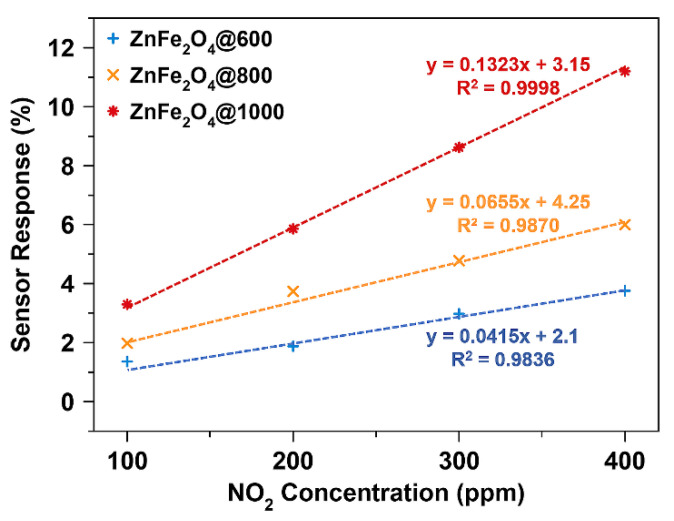
The calibration curves for different ZnFe_2_O_4_ samples showing NO_2_ sensitivity of the chemiresistive devices.

Figure 7 shows the calibration curves obtained by plotting the maximum gas response of the annealed ZnFe_2_O_4_ nanoparticles as a function of gas concentration. All ZnFe_2_O_4_ samples exhibit a linear response in the concentration range of 100–400 ppm as demonstrated by the straight lines in Figure 7. The sensitivity of ZnFe_2_O_4_-based chemiresistive devices can be calculated from the slope of a straight line. The sensitivity of NO_2_ sensors follows the order: ZnFe_2_O_4_@1000 > ZnFe_2_O_4_@800 > ZnFe_2_O_4_@600, which describes the effect of annealing temperature on sensor performance. An increase in annealing temperature improves the NO_2_ sensing properties of ZnFe_2_O_4_ nanoparticles. Therefore, ZnFe_2_O_4_@1000 nanoparticles exhibit 2.0-fold and 3.2-fold high sensitivity compared to ZnFe_2_O_4_@800 and ZnFe_2_O_4_@600 nanoparticles, respectively.

**Figure 8:**
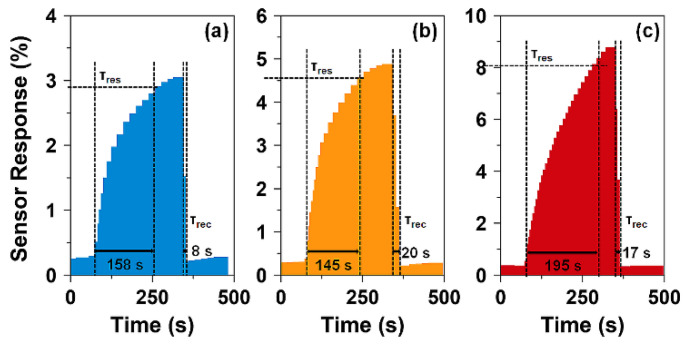
The response and recovery times of ZnFe_2_O_4_ nanoparticles annealed at (**a**) 600, (**b**) 800, and (**c**) 1000 °C calculated from their respective responses to 300 ppm NO_2_.

Figure 8 shows the kinetics of ZnFe_2_O_4_-based chemiresistive gas sensors. The response (*τ_res_*) and recovery (*τ_rec_*) times of the annealed ZnFe_2_O_4_ nanoparticles are estimated from their response to 300 ppm NO_2_ gas. All samples show fast response and recovery times. The response times are in the range of 145–195 s and follow the order: ZnFe_2_O_4_@800 > ZnFe_2_O_4_@600 > ZnFe_2_O_4_@1000. Thus, ZnFe_2_O_4_@1000 nanoparticles exhibit slightly longer response (*τ_res_*) times compared to ZnFe_2_O_4_@600 and ZnFe_2_O_4_@800 nanoparticles. The recovery times are sharp (i.e., ≤20 s) for all samples and all sensors exhibit 100% recovery to their original state. At elevated temperatures, the recovery times are generally faster [10]. Overall, ZnFe_2_O_4_@1000 nanoparticles exhibit excellent NO_2_ gas sensing properties such as high sensitivity, good response kinetics, and linear response in the tested concentration range.

**Figure 9:**
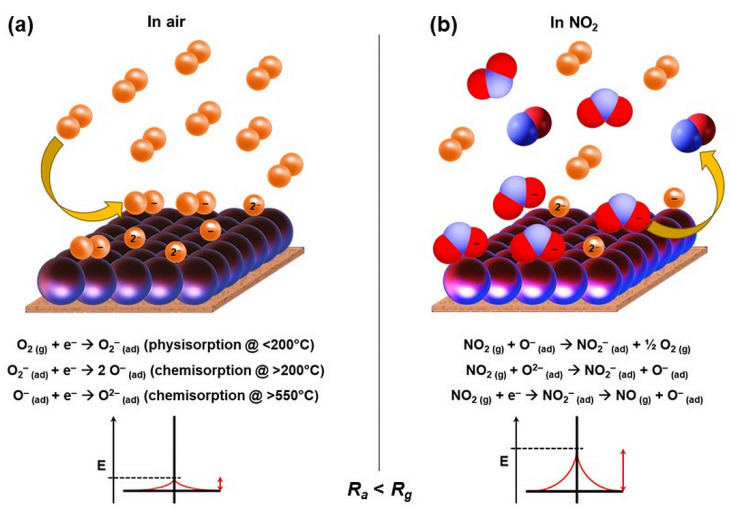
The gas sensing mechanism of ZnFe_2_O_4_ nanoparticles-based chemiresistive gas sensors.

The chemiresistive gas sensors function on the principles of changes in resistance of the sensing element when test gas molecules interact with the semiconductor surface [9]. Figure 9 demonstrates the gas sensing mechanism of ZnFe_2_O_4_ nanoparticles. ZnFe_2_O_4_ is an *n*-type semiconductor [56]. In principle, when the ZnFe_2_O_4_-based chemiresistive device is exposed to air at elevated temperatures, active oxygen species are adsorbed on the surface of ZnFe_2_O_4_ nanoparticles. As shown in Figure 9a, O_2_ molecules are physisorbed (O_2_^−^) at low temperatures (<200 °C) and subsequently chemisorbed (O^–^ and O^2–^) at elevated temperatures (>200 °C) by capturing mobile electrons (e^–^) from the surface. This leads to the formation of a charge depletion layer on the surface of ZnFe_2_O_4_ nanoparticles. Afterward, the surface is exposed to different concentrations of NO_2_ gas and NO_2_ being an electron-withdrawing molecule [57] further extracts mobile e^−^ from the surface or interacts with the chemisorbed oxygen species, as shown in Figure 9b. Consequently, the density of major charge carriers (e^−^) decreases, and the thickness of the depletion region increases, which increases the resistance of the device. The redox reactions taking place on the surface of ZnFe_2_O_4_ nanoparticles are depicted in Figure 9.

Considering the mechanism described above, it is important to understand the behavior of ZnFe_2_O_4_ nanoparticles annealed at different temperatures. It is believed that semiconducting metal oxides with more oxygen vacancies adsorb a large number of active oxygen species, which in turn facilitates the surface redox reactions with the target gas molecules and improves the gas response [55]. Thus, oxygen vacancies and lattice defects play a major role in determining the gas response of ZnFe_2_O_4_ nanoparticles. Therefore, ZnFe_2_O_4_@1000 nanoparticles exhibit the best NO_2_ gas sensing properties despite their slightly bigger crystallite size and smaller specific surface area. Table 3 shows a comparison of ferrite-based chemiresistive NO_2_ sensors. The results demonstrate the potential of stable ZnFe_2_O_4_@1000 nanoparticles for high-temperature gas sensing applications.

## 4. Conclusions

In summary, this study presents the effects of annealing temperature on the microstructure evolution and gas sensing properties of ZnFe_2_O_4_ nanoparticles. A high-energy ball-milling procedure is used to prepare pure ZnFe_2_O_4_ nanoparticles that are annealed at different temperatures (600–1000 °C). ZnFe_2_O_4_ nanoparticles exhibit a random spinel lattice structure that is distorted during high-temperature annealing. The XRD results show an increase in the crystallite size, but a reduction in the lattice parameter and volume that is attributed to the presence of lattice defects as oxygen vacancies. The oxygen vacancies play a major role in controlling the sensitivity of ZnFe_2_O_4_ nanoparticles. Thus, ZnFe_2_O_4_@1000 nanoparticles (annealed at 1000 °C) reveal the superior gas sensing properties with the highest sensitivity, good response kinetics, and linear response toward 100–400 ppm NO_2_ gas. This is the first example of a ZnFe_2_O_4_@1000-based chemiresistive device showing significant gas response and stable sensor performance at 600 °C.

## Figures and Tables

**Table 1:** The structural properties of ZnFe_2_O_4_ nanoparticles, annealed at different temperatures (T).

Sample	T (°C)	D (nm)	a (Å)	V (Å^3^)	d_311_ (Å)	ρ_xrd_ (g/cm^3^)	S (m^2^/g)
BM-ZnFe_2_O_4_	-	9.30	8.445	602.3	2.546	5.321	121.24
ZnFe_2_O_4_@600	600	18.71	8.430	599.0	2.542	5.350	59.94
ZnFe_2_O_4_@800	800	23.03	8.424	597.8	2.540	5.361	48.59
ZnFe_2_O_4_@1000	1000	23.25	8.420	597.0	2.539	5.368	48.07

**Table 2:** The chemical composition of ZnFe_2_O_4_ nanoparticles annealed at different temperatures.

Sample	Zn		Fe		O	
	(wt.%)	(at.%)	(wt.%)	(at.%)	(wt.%)	(at.%)
ZnFe_2_O_4_@600	26.61	14.08	47.15	29.17	26.24	56.75
ZnFe_2_O_4_@800	25.60	13.51	48.04	29.64	26.36	56.85
ZnFe_2_O_4_@1000	25.65	13.68	48.67	30.34	25.69	55.98

**Table 3:** A comparison of the sensing properties of chemiresistive-type NO_2_ gas sensors.

Material	Fabrication Method	Temperature (°C)	Detection Range (ppm)	Response ^†^ (S)	Response Time (s)	Recovery Time (s)	Reference
CuFe_2_O_4_	Coprecipitation	27	20–240	72%	8	5	[58]
ZnFe_2_O_4_	Hydrothermal	125	1–10	248 ^‡^	6.5	11	[59]
Pd-doped BiFeO_3_	Sol-gel	140	50–3500	93%	60	100	[60]
CoFe_2_O_4_	Spray pyrolysis	150	20–80	95%	5	114	[61]
ZnO/ZnFe_2_O_4_	Wet chemical	200	0.1–20	~ 300 ^‡^	7	15	[25]
Cu-doped α-Fe_2_O_3_	Electrospinning	300	5–50	2 ^‡^	118	258	[62]
ZnFe_2_O_4_	Ball-milling	600	100–400	11%	195	17	This work
Sb-doped Zn_2_SnO_4_	Sputtering	600	50–300	~ 4 ^‡^	-	-	[63]

^†^ The response (S) is reported for the highest tested concentration of NO_2_ gas: *S(%) = (R_g_ – R_a_) ×* 100/*R_a_*. ^‡^ If not reported as S(%), the response is measured as: *S = R_g_/R_a_*.
